# Evolution of Computerized Provider Order Entry Documentation at a Leading Tertiary Care Referral Center in Riyadh

**DOI:** 10.3390/healthcare14020179

**Published:** 2026-01-10

**Authors:** Hanan Sabet Alanazi, Yazed Alruthia

**Affiliations:** 1 Prince Mohammed Bin Abdulaziz Hospital, Riyadh 14214, Saudi Arabia; alanazihan@pmah.med.sa; 2 Department of Clinical Pharmacy, College of Pharmacy, King Saud University, Riyadh 11451, Saudi Arabia

**Keywords:** computerized provider order entry, patient safety, electronic health record, electronic health record data, quality improvement, Saudi Arabia

## Abstract

**Background**: Computerized Provider Order Entry (CPOE) systems are critical for medication safety, but their effectiveness relies heavily on the completeness of entered data. Incomplete clinical and anthropometric information can disable Clinical Decision Support Systems (CDSSs), compromising patient safety. **Objective**: This study aimed to assess the longitudinal evolution of CPOE data completeness, specifically focusing on “Breadth Completeness” (the presence of essential clinical variables), and to identify factors predicting data integrity in a tertiary care setting. **Methods**: A retrospective cross-sectional study was conducted at a 500-bed tertiary referral center in Riyadh. Data were extracted from the Cerner Millennium CPOE system for three “steady-state” years (2015, 2017, and 2019); years involving major system overhauls (2016 and 2018) were excluded to avoid structural bias. A total of 600 unique patient encounters (200 per year) were selected using systematic random sampling from a chronologically ordered sampling frame to minimize temporal bias. The primary outcome was “Breadth Completeness,” defined as the presence of eight key variables: age, gender, marital status, weight, height, diagnosis, vital signs, and allergies. Secondary outcomes included documentation consistency (daily notes). Multivariable logistic regression, adjusted for potential confounders, was used to determine predictors of completeness. **Results**: The rate of primary data completeness (Breadth) improved significantly over the study period, rising from 5.5% in 2015 to 26% in 2017 and 49.5% in 2019. In the multivariable analysis, the year of documentation (OR = 17.47 for 2019 vs. 2015, *p* < 0.0001) and length of hospitalization (OR = 1.04, *p* = 0.045) were significant predictors of completeness. Pharmacist-led medication reconciliation was associated with a 2.5-fold increase in data completeness in bivariate analysis (*p* < 0.0001). **Conclusions**: While system maturity has driven substantial improvements in CPOE documentation, critical gaps persist, particularly in anthropometric data required for safety alerts. The study underscores the necessity of mandating “hard stops” for core variables and formalizing pharmacist involvement in data reconciliation to ensure patient safety.

## 1. Introduction

The primary objective of healthcare practices, as recommended by the World Health Organization (WHO), is to ensure the provision of quality and safe patient care to every individual. Achieving this standard requires hospital administrators and healthcare providers to maintain strict accountability for accurate and thorough documentation within each patient’s medical profile. The demands for completeness and precision in entered data are exceptionally high as they are crucial for effective clinical decision-making, patient safety, and high-quality outcomes [1].

A pivotal measure in enhancing patient safety is the implementation of Computerized Provider Order Entry (CPOE) systems, which are crucial in minimizing Adverse Drug Events (ADEs) and medication errors [2,3]. Medication errors are a significant public health concern; annually, between 7000 and 9000 individuals in the United States succumb to them, and studies suggest that ADE incidents in adult hospitals range from 2% to 7% per 100 admissions [4,5]. Alarmingly, a significant portion (28%) of ADEs stem from preventable medication errors, of which 56% are a direct result of issues in the drug ordering process [6]. Research has highlighted the risks posed by illegibility and incompleteness in traditional paper-based medication orders, with findings revealing that over 20% of orders are either unreadable or contain illegible prescriptions [7,8]. Therefore, CPOE systems represent a significant step toward improving patient safety by substantially reducing order-related errors compared to traditional methods [4]. These orders can include medications, laboratory tests, radiological requests, and others, which can be entered by physicians, pharmacists, laboratory technicians, and other healthcare professionals [9]. When combined with Clinical Decision Support Systems (CDSSs), CPOE offers features that actively drive data integrity through functional dependency. A prime example in our setting is the standardized dose reminder for antimicrobial stewardship. These systems often utilize algorithms (e.g., Cockcroft–Gault) to recommend renal dosing for antibiotics. However, these algorithms cannot function if the patient’s weight, height, and creatinine levels are missing from the CPOE fields. Consequently, the CDSSs alert acts as a forcing function: to receive the dosing guidance and proceed with the order, the clinician is compelled to enter the missing anthropometric data, thereby directly improving the ‘breadth’ of the patient record [10,11]. The data generated through CPOE is vital not only for direct patient care but also for health policy planning, quality improvement, service enhancement, and crucial clinical research endeavors [12].

The integrity of collected data, particularly its accuracy and completeness, is crucial for several reasons: it facilitates appropriate patient treatment, prevents medication errors, accurately estimates disease prevalence, ensures compliance with established healthcare standards and guidelines, and supports comprehensive clinical research [13]. In the context of Electronic Health Record (EHR) systems, data quality is characterized by attributes such as legibility, accuracy, completeness, and meaning [8,14,15]. Although CPOE systems are designed to enhance these attributes, research has revealed persistent inaccuracies and incompleteness in clinical documentation, including medication allergy records, which can undermine the primary objective of reusing clinical data [2,15]. Various factors have been identified as potential drivers of such incomplete documentation [16,17,18]. High turnover rates among healthcare providers, particularly the reliance on new residents or temporary (locum) staff, may contribute to inconsistent adherence to documentation protocols. This is often compounded by varying levels of training and familiarity with the CPOE system. For instance, a study in Korea reported that only 27.4% of medical records were fully complete, attributing this to workflow pressures and inadequate training [17,19]. Similarly, research from Jeddah identified staff turnover and training deficits as significant barriers to complete documentation [16]. Additionally, the phenomenon of “alert fatigue”—where excessive or irrelevant alerts cause users to ignore safety notifications—remains a documented challenge that can diminish the utility of the system [13]. For a database to be deemed suitable for its intended purpose, its completeness must be formally structured and documented [18]. In the specific context of a tertiary referral center for infectious diseases (e.g., MERS-CoV, COVID-19), the definition of ‘data integrity’ extends beyond standard administrative requirements. Here, Weiskopf’s dimension of ‘breadth’ must be operationalized to include epidemiological contact history, isolation levels, and precise anthropometric data [20]. For example, in the management of highly contagious pathogens, a medical record is only functionally ‘complete’ if it contains the data necessary to trigger automated isolation protocols and calculate weight-based dosing for anti-infective agents with narrow therapeutic indices. Missing data in this context does not merely represent a documentation gap but a potential breach in infection control and patient safety. Weiskopf et al. identified four measurable definitions of data completeness [21], which include the following:

1. Documentation: The record contains all clinical observations made during a patient encounter.
2. Breadth: The record contains all desired types of data relevant to the clinical area.

Despite the widespread implementation of CPOE systems across Saudi Arabia, concerns about data completeness persist. Recently, the landscape has evolved significantly with the Saudi Ministry of Health’s introduction of the National Health Information Exchange (nHiE) Policy in 2023 and the Open Data Policy (2024), which enforce rigorous standards for data quality to enable interoperability [22]. These regulations emphasize that data must not only be present but also standardized to support advanced initiatives such as the Seha Virtual Hospital, which was launched in 2022 [23]. However, achieving these standards remains challenging; recent local studies by Al-Jafar et al. (2023) and Alhur (2023) highlight that while infrastructure has improved [24,25], user adherence to documentation protocols—particularly in high-pressure environments—remains a critical bottleneck [24,25,26]. For instance, studies conducted in the Western Region (Jeddah) have primarily examined documentation barriers and quality within primary healthcare settings, while others have concentrated on specific metrics such as prescription legibility rather than holistic data completeness [16]. These existing inquiries often overlook the dynamic evolution of data quality that occurs as systems mature. There is a distinct lack of longitudinal analysis regarding how data integrity changes over time within major tertiary care referral centers in the Central Region (Riyadh) [7]. Therefore, this study distinguishes itself from previous regional research by shifting focus from immediate implementation challenges to the long-term sustainability of data quality, aiming to fill the academic gap regarding the temporal progress of CPOE documentation in the Saudi healthcare landscape.

## 2. Methods

### 2.1. Study Design, Setting, and Duration

This was a retrospective cross-sectional study utilizing data retrieved from the CPOE database at Prince Mohammad bin Abdulaziz Hospital in Riyadh, Saudi Arabia. The hospital is a 500-bed tertiary care referral center specializing in the treatment of patients with highly contagious viral or bacterial infections, including Middle East Respiratory Syndrome Coronavirus (MERS-CoV) and Coronavirus Disease 2019 (COVID-19), who were transferred from various facilities across the central region and the country.

The CPOE system, Cerner Millennium, was adopted by the hospital in April 2014 and fully implemented in January 2015 following comprehensive training for information technology personnel and subsequent educational sessions for medical staff. The study period spanned from January 2015 until June 2019. Data from the years 2016 and 2018 were excluded to isolate long-term documentation trends from temporary implementation artifacts. During these periods, the hospital underwent significant disruptive technical transitions that rendered the data incomparable to ‘steady-state’ years. Consistent with the ‘productivity dip’ phenomenon described in health informatics literature [27,28], these transition periods are characterized by temporary erratic documentation behavior as users navigate new workflows. Therefore, to assess the true maturation of data quality rather than transient adoption struggles, analysis was restricted to the stabilized periods of 2015 (baseline), 2017 (post-optimization), and 2019 (mature usage).

### 2.2. Ethical Approval

The study received ethical approval on 15 March 2020, from the Institutional Review Board (IRB) of the Central Second Health Cluster, King Fahd Medical City (Approval number: 20-129E). The data were anonymized, and no personal identifiers were collected. The study adhered to the ethical principles outlined in the Helsinki Declaration for clinical research [29].

### 2.3. Sample Size and Sampling Technique

#### 2.3.1. Sample Size Determination

The minimum required sample size was estimated to be 571 patients. This was calculated for logistic regression with a binomial distribution using the G*Power^®^ software version 3.1, assuming an odds ratio of 2 (hypothesizing an almost double chance of having complete CPOE data annually) at an alpha (α) of 0.05 and a power (1-β) of 0.95. To ensure sampling adequacy and exceed the minimum requirement, a total of 600 patient records were selected, with 200 patients randomly selected from each of the sampled years (2015, 2017, and 2019).

#### 2.3.2. Sampling Technique and Population

Patient records were randomly recruited from the local citizens and residents of the east side of Riyadh who visited the hospital during the study period (January 2015–June 2019). While the facility accepts referrals, the study population primarily consists of residents from the east side of Riyadh, reflecting the hospital’s primary geographic catchment area. To prevent seasonality bias, the sampling frame for each year was first ordered chronologically by admission date (1 January to 31 December). We then applied a systematic random sampling technique, selecting every 10th record. This method acted as a proxy for temporal stratification, ensuring proportional representation of all months and seasons. The unit of analysis was the unique patient encounter; duplicate records for the same patient were removed to ensure statistical independence. To ensure the rigor of the data extraction process and minimize subjective assessment bias, a data consistency check was implemented. While the primary investigator reviewed all selected records, the co-investigator (research adviser) performed an independent validation on a random subset of the data (representing 10% of the total sample). Inter-rater reliability was assessed to determine the consistency of the “completeness” classification between the two reviewers. Any discrepancies identified during this cross-checking process were resolved through consensus discussion to ensure strict adherence to the data completeness definitions prior to the final analysis.

### 2.4. Inclusion and Exclusion Criteria

All inpatient encounters (Intensive Care Unit, Operating Room, Internal Medicine, and External Medicine) and outpatient encounters (Emergency Room, Day Care Surgery, and Outpatient Clinic) for randomly selected records from the years 2015, 2017, and 2019 were included in the study.

### 2.5. Data Collection and Variables

A standardized data collection sheet was created to facilitate the extraction of relevant variables from the EHR database. The purpose of the data collection was to assess the completeness of CPOE data over the three sampled years (2015, 2017, and 2019). The selection of variables was guided by Weiskopf et al.’s definition of “breadth” (data relevant to the clinical area) [20]. Although the study setting is a tertiary center specializing in infectious diseases (MERS-CoV and COVID-19), the variables prioritized for this study were those essential for the foundational functionality of the CPOE system and its associated CDSSs. Anthropometric data (height and weight) and clinical baselines (vital signs and allergies) were prioritized over infectious disease-specific indicators (such as contact history or isolation status) because they are critical prerequisites for medication safety mechanisms, including automated dosage calculations, renal function adjustments, and drug–drug interaction checks. Without these core data points, the CPOE system cannot effectively reduce Adverse Drug Events (ADEs), which is the primary safety objective of the system.

To ensure precise measurement, “Data Completeness” was operationalized using two distinct definitions adapted from Weiskopf et al. [20]:

1. **Primary Outcome (Breadth Completeness)**: The primary measure of completeness was defined as the presence of valid data across eight essential clinical variables: age, gender, marital status, weight, height, diagnosis, vital signs, and allergies. A record was coded as “Complete” (1) only if all eight fields contained data; otherwise, it was “Incomplete” (0).
2. **Secondary Outcome (Documentation Completeness)**: As an exploratory measure, we assessed longitudinal consistency, defined as the presence of a clinical narrative note for every day of the patient’s admission.
3. **Data Plausibility**: To ensure data validity, biological plausibility limits were applied during cleaning. Values falling outside accepted ranges (e.g., Weight: 10–300 kg; Height: 50–250 cm; Heart Rate: 30–250 bpm) were flagged as measurement errors and treated as missing data.

The following variables were collected:

- Demographic characteristics: Gender, marital status, age, weight, height, diagnosis, vital signs, and allergies.
- Anthropometric variables, specifically weight and height, were included as they are fundamental for the CPOE system’s CDSS capabilities, particularly for accurate weight-based medication dosing and nutritional assessment.
- Alert notification information: Type of alert.
- Medications information: History of medication reconciliation and number of medications ordered.
- Clinical documentation: Length of hospitalization (LOS) with narrative notes through different encounter types (e.g., inpatient or outpatient).

The purpose of the data collection was to assess the completeness of CPOE data over the three sampled years. The data from 2015 served as the baseline measurement for this study, representing the status of documentation immediately following the system’s full implementation in January 2015.

### 2.6. Statistical Analysis

Given that the primary objective of this study was to evaluate the completeness of documentation, missing data points within the electronic health records were treated as the outcome of interest rather than statistical errors. Therefore, no statistical imputation methods (e.g., mean imputation) were applied. Missing values were coded as “Incomplete” for binary outcome variable. Descriptive statistics, including means and standard deviations (SDs) for continuous variables, and frequencies and percentages for categorical variables, were used to summarize the findings across the sampled years.

Normality of continuous variables was assessed using the Shapiro–Wilk test and visual inspection of histograms. For variables violating normality assumptions, non-parametric tests (Kruskal–Wallis) were employed; otherwise, one-way Analysis of Variance (ANOVA) was used. Categorical data were compared using the Chi-square test, with effect sizes reported using Cramer’s V. To minimize information loss, Length of Stay (LOS) was analyzed as a continuous variable within the regression model, following verification of the linearity of the logit. The variable ‘Year’ was treated as categorical variable to avoid assumptions of linear progression. The variable ‘Alert Notifications’ was excluded from the regression model to avoid endogeneity, as alerts mechanically compel data entry. Model discrimination was evaluated using the c-statistic (AUC), and calibration was assessed using the Hosmer–Lemeshow goodness-of-fit test and calibration plots. The completeness of each included variable was compared across the three sampled years (2015, 2017, and 2019).

1. To examine the factors associated with the primary outcome, which was the odds of a patient record having complete data (a binary outcome: Complete/Incomplete), a logistic regression analysis was conducted. To control for multiplicity arising from multiple bivariate comparisons, a Bonferroni correction was applied whenever appropriate. Bivariate Logistic Regression: This was performed to assess the unadjusted association between the outcome (data completeness) and several key predictor variables:
  o Complete documentation of medication reconciliation.
  o Length of stay (LOS) in the hospital.
  o Type of patient encounter (inpatient or outpatient).
  o Inclusivity of data due to alert systems.
  o Number of medications ordered.
  o Year of observation (2015, 2017, or 2019).
2. Multiple Logistic Regression: A final multivariable model was constructed using a purposeful selection strategy. Variables were chosen based on (1) statistical significance or potential association in the bivariate analysis (set at a liberal threshold of *p* < 0.10 to prevent missing confounders), and (2) clinical relevance [20]. Specifically, demographic characteristics such as age and gender were forced into the final model regardless of their bivariate significance to strictly control for demographic differences between the yearly cohorts. This model helped identify the factors that independently influenced data completeness while controlling for potential confounders.
3. To assess whether the improvement in data completeness over time differed between clinical settings, a logistic regression model including an interaction term between ‘Year’ and ‘Encounter Type’ (Inpatient vs. Outpatient) was constructed. A *p*-value of <0.05 for the interaction term was considered indicative of a significant difference in the rate of improvement.
4. Sensitivity Analysis: Given the low event rate (EPV) in the 2015 cohort (5.5% completeness), Firth’s penalized logistic regression was conducted as a sensitivity check to reduce small-sample bias.
5. Multicollinearity among independent variables was assessed using the Variance Inflation Factor (VIF), with a threshold of <2.5 indicating negligible collinearity. plausible interaction terms (e.g., Year × Length of Stay) were explored but were excluded from the final model as they did not reach statistical significance (*p* > 0.05). The goodness-of-fit for the final logistic regression model was evaluated using the Hosmer–Lemeshow test and the c-statistic (Area Under the Curve) to assess calibration and discrimination, respectively.

**Figure 1:**
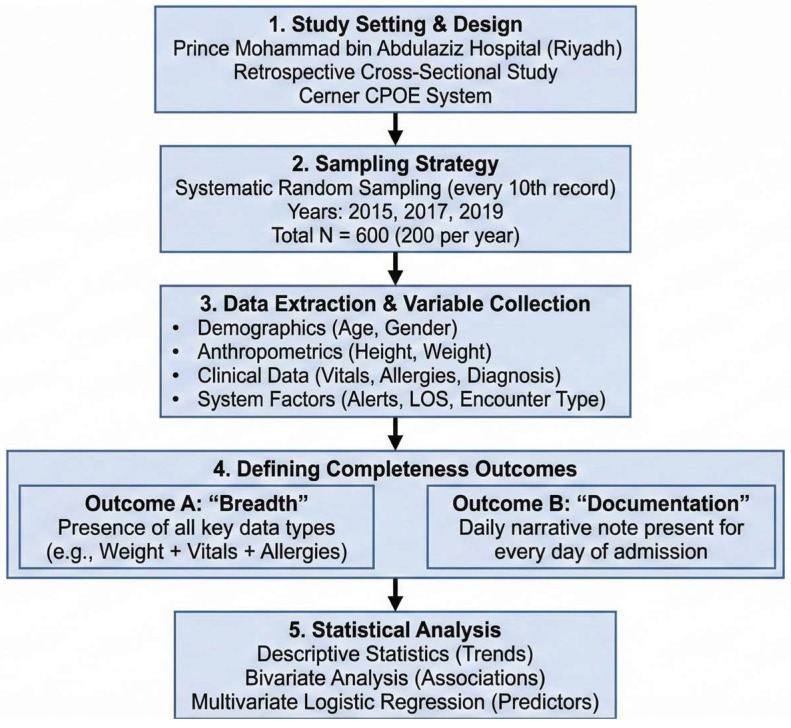
Visual representation of the study methodology.

All statistical analyses were performed using the SAS^®^ software, version 9.4 (SAS Institute Inc., Cary, NC, USA). Statistical significance was set at a two-sided *p*-value of <0.05. Figure 1 presents a visual representation of the study design.

## 3. Results

### 3.1. Baseline Characteristics

**Figure 2:**
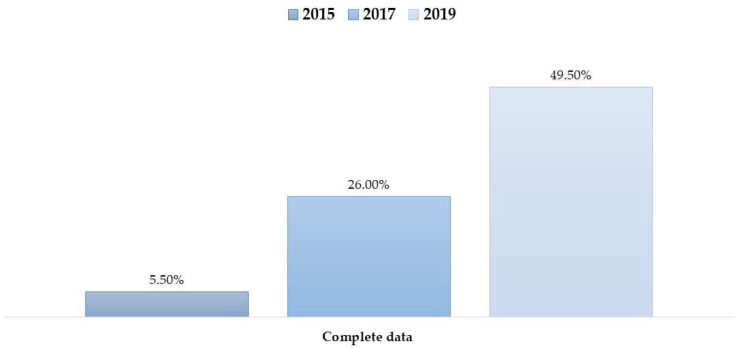
Percentage of patient records meeting the “Breadth” completeness definition (presence of all key variables) across the study years (2015, 2017, 2019).

A total of 600 patients, distributed evenly across the three study years, were selected (n = 200 for each year (2015, 2017, and 2019)). The patients’ baseline characteristics are summarized in Table 1. There was a statistically significant difference in the mean age of patients across the recruitment years (*p* < 0.0001). Patients recruited in 2019 were significantly older (mean ± SD: 50.38 ± 16.13 years) than those recruited in 2017 (46.46 ± 16.93 years) and 2015 (38.62 ± 18.59 years) (see Figure 2 for distribution). Regarding gender, the majority of patients were male (>50%) across all years, with no significant difference observed between the yearly cohorts (*p* = 0.806). The percentage of married patients was significantly different across the years (*p* = 0.0005), with the highest percentage observed in the 2019 cohort (76.5%) compared to 2017 (74.0%) and 2015 (60.0%). The mean number of prescription medications showed a significant difference across the years (*p* < 0.0001). Patients recruited in 2015 had a statistically significant higher number of prescription medications (mean ± SD: 5.53 ± 2.62) compared to those recruited in 2019 (5.05 ± 2.18) and 2017 (4.58 ± 2.57) (*p* = 0.0007).

### 3.2. Completeness of CPOE Data Based on Different Definitions

#### 3.2.1. Breadth of Data

Data for demographic variables (age, gender, and marital status) were complete (100%) across the study population. However, varying degrees of missingness were observed in clinical and anthropometric variables. To address data quality transparency, the frequency and proportion of missing data for each variable are detailed in Table 2. As shown in Table 2 and Tables S1 and S2, a significant reduction in missing data was observed over the study period. For instance, the proportion of missing weight measurements decreased from 74.0% in 2015 to just 7.0% in 2019. Similarly, missing documentation for allergies dropped from 85.0% to 27.0% over the same period.

Despite these improvements in individual fields, the number of patients with complete data across all variables (i.e., no missing observations overall) remained relatively low, though it also improved over time (Figure 2).

#### 3.2.2. Complete Documentation

**Figure 3:**
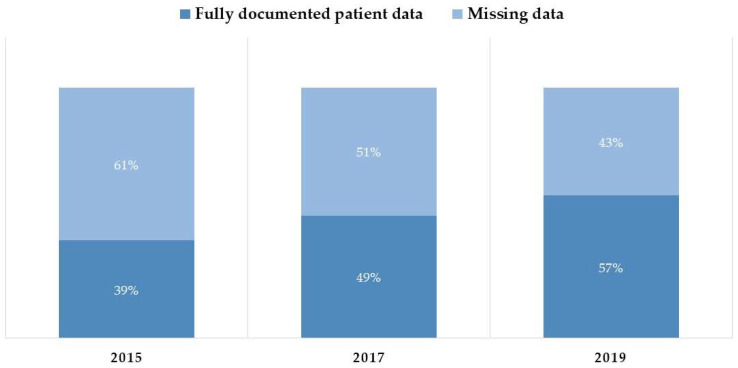
Proportion of patient records meeting the “Documentation” completeness definition (daily narrative notes) versus records with missing daily notes.

The completeness of data documentation, defined as the entry of notes for all patient admission days into the CPOE system, improved significantly over the study period. Specifically, the completeness rate rose from 39% in 2015 to 49% in 2017, and further to 57% in 2019. The progressive improvement in complete data based on this documentation definition across the three years is presented visually in Figure 3.

#### 3.2.3. CPOE Data Completeness Trends

**Figure 4:**
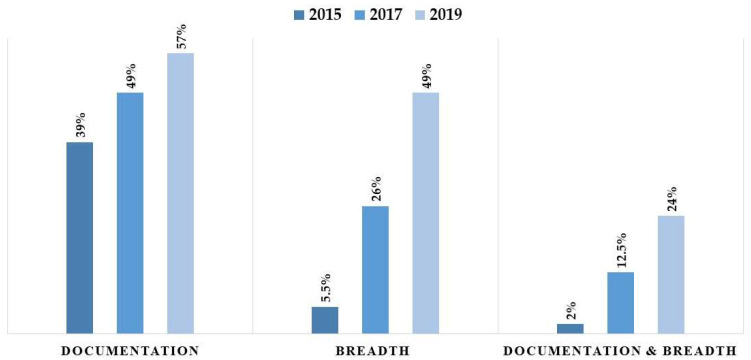
Comparison of data completeness rates using “Documentation,” “Breadth,” and “Combined” definitions.

**Figure 5:**
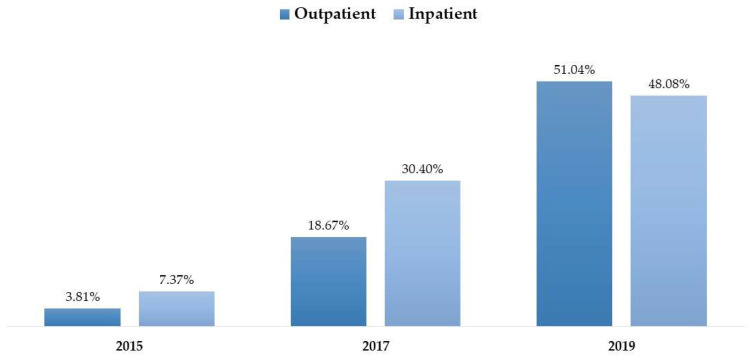
Comparative trends in data completeness stratified by clinical setting (Inpatient vs. Outpatient).

The progress in CPOE data completeness was examined over the studied years using different definitions. Completeness showed an increase when applying the breadth definition, the documentation definition, or when applying both definitions combined. When completeness was examined using both definitions, the proportion of complete data reached 24% in 2019 (see Figure 4). While Figure 5 illustrates numerical differences in completeness trends—with Inpatient settings showing higher completeness in 2017 (30.40% vs. 18.67%) but lower in 2019 (48.08% vs. 51.04%)—formal statistical testing was conducted to verify these divergences. The logistic regression interaction analysis (Year × Encounter Type) yielded a *p*-value of 0.182. This demonstrates that the temporal improvement in documentation quality was similar across both settings, despite the variations observed in the unadjusted proportions. Likewise, the proportion of complete data rose from 3.81% in 2015 to 51.04% in 2019, with the difference being statistically significant (*p* < 0.0001).

#### 3.2.4. Bivariate Association of Variables with Data Completeness

To examine the association between various study variables and overall data completeness, a bivariate logistic regression analysis was performed. The variables included (1) year of documentation, (2) length of stay (LOS), (3) pharmacist medication history reconciliation, (4) type of patient encounter, (5) presence of alert notification, and (6) number of medications (Table 3).

The individual variable findings were as follows:

1. **Year of Documentation**: Year of documentation was a statistically significant predictor. Compared to the baseline year of 2015, the odds of data completeness increased 6-fold in 2017 (OR = 6.04, 95% CI: 3.04–11.98) and nearly 17-fold in 2019 (OR = 16.84, 95% CI: 8.63–32.85) (Table 3).
2. **Length of Stay (LOS)**: Length of stay modeled as a continuous variable was also significant (OR = 1.04, 95% CI: 1.01–1.06, *p* = 0.0023) (Table 3).
3. **Pharmacist Medication History Reconciliation**: Medication reconciliation status showed a highly significant effect on CPOE data completeness across the three years (*p* < 0.0001). Patients who underwent medication reconciliation had 2.51-times greater odds of having complete CPOE data (OR = 2.51, 95% CI: 1.73–3.64, *p* < 0.0001) (Table 3). Furthermore, compliance with medication reconciliation documentation improved dramatically from 1% completeness in 2015 to 57% completeness in 2019, averaging 35% over the study period. This process was performed by clinical pharmacists.
4. **Type of Patient Encounter**: Although a difference in completeness was observed between inpatient (52% complete in 2019) and outpatient (48% complete in 2019) encounters (*p* = 0.009), the bivariate logistic regression found no statistically significant association between the type of patient encounter (inpatient vs. outpatient) and the overall CPOE data completeness (*p* = 0.165).
5. **Presence of Alert Notification**: The presence of an alert notification demonstrated a significant effect on CPOE completeness across all study years (*p* < 0.0001). Specifically, 70% of records categorized as complete had at least one corresponding alert notification.
6. **Number of Medications**: Based on the breadth definition of completeness, there was no statistically significant association between the number of prescribed medications and data completeness (*p* = 0.060). However, patients prescribed more than three medications showed a higher fulfillment rate (64.9%) compared to those with three or fewer medications.

### 3.3. Predictors of CPOE Data Completeness

**Figure 6:**
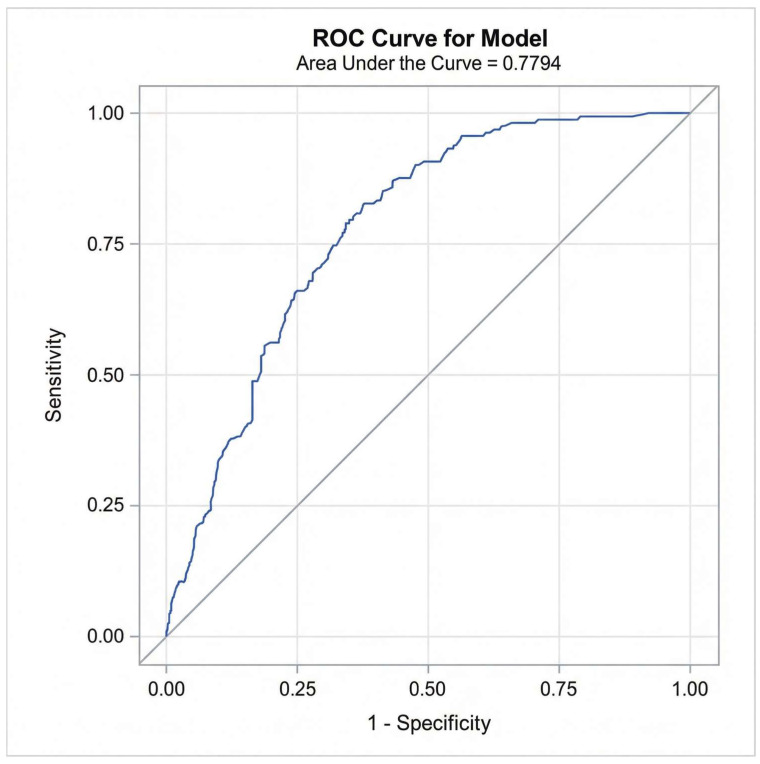
Receiver Operating Characteristic (ROC) curve for the final multivariable logistic regression model predicting CPOE data completeness.

**Figure 7:**
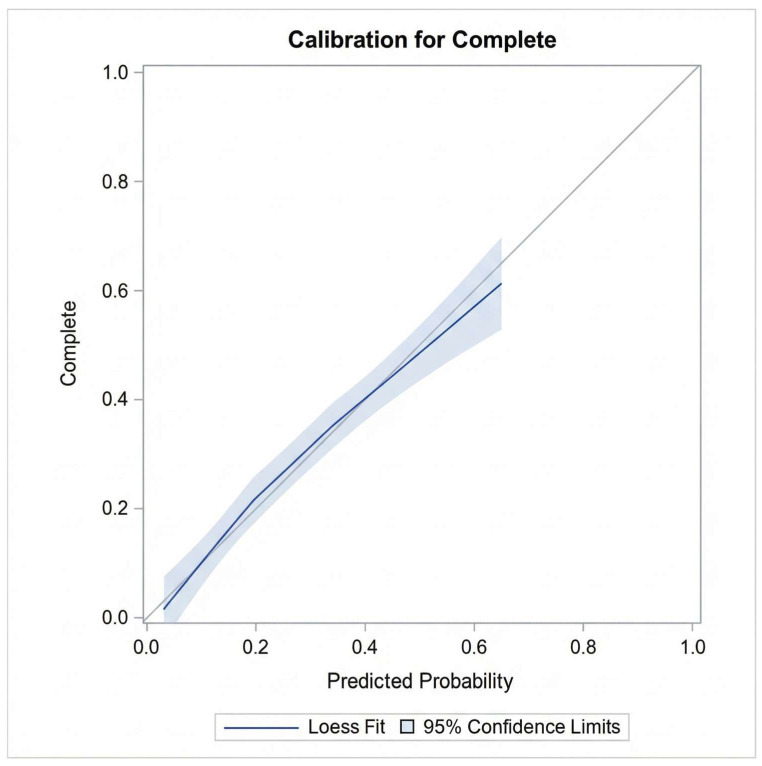
Calibration plot comparing predicted probabilities of data completeness against observed outcomes.

In the multiple logistic regression analysis, only time progression (year) and length of stay/hospitalization remained significantly associated with CPOE data completeness after controlling for age, gender, number of medications, medication reconciliation, and type of hospital admission (e.g., inpatient encounter) (see Table 4). Diagnostic checks confirmed the absence of multicollinearity (Max VIF < 1.5). The variable ‘Alert Notifications’ was excluded from this final model to avoid endogeneity bias. Model performance was rigorously evaluated using both discrimination and calibration metrics. The model demonstrated acceptable discrimination with a c-statistic (AUC) of 0.78 (95% CI: 0.74–0.82) (Figure 6). Calibration was assessed using the Hosmer–Lemeshow goodness-of-fit test, which indicated adequate fit (Chi-square = 7.08, *p* = 0.527). This was visually confirmed by the calibration plot (Figure 7), where the Loess smoothed line of predicted probabilities closely tracked the ideal 45-degree diagonal, demonstrating strong agreement between the model’s predicted risks and the observed data completeness rates.

## 4. Discussion

This study assessed the completeness of CPOE data over a five-year period, revealing a significant, albeit gradual, improvement in documentation practices. The primary finding indicates that data completeness, when defined by the presence of eight key variables (breadth) and daily documentation, increased from a mere 2% in 2015 to 12.5% in 2017, reaching 24% in 2019. This positive trend supports our research hypothesis that CPOE data completeness improves over time as health systems mature and users gain proficiency.

The definition of “completeness” profoundly influenced the outcomes. When a less stringent definition was applied, requiring a note for each day of the patient’s admission, the completeness rate rose to 57%. This highlights a critical challenge in health informatics research: the lack of a standardized definition for data completeness, which can lead to wide variations in reported outcomes and complicate comparisons across studies [21]. Our findings, using a multi-variable breadth definition, showed a 49% completeness rate in 2019. This is comparable to, though lower than, that from a study by Alwhaibi et al., which reported 83.1% completeness based on seven variables [30]. The discrepancy likely stems from the different variables included in our analysis, such as vital signs, weight, and height, which were frequently missing in our dataset. The most commonly incomplete fields were vital signs (57.2% missing), height (53.2%), allergies (52.3%), and weight (46.8%), indicating systemic issues in data capture for these fundamental clinical parameters.

### 4.1. Factors Influencing Data Completeness

Several factors were identified as potential drivers of incomplete CPOE documentation, such as the high turnover rate among healthcare providers. This is often compounded by varying levels of training and familiarity with the CPOE system [15,19]. The findings of our study imply that there are staff training deficits, and these findings align with those of Alghamdi et al. [16], who identified training deficits as a primary barrier to data quality in Saudi primary healthcare centers. However, our analysis reveals a divergence in influencing factors dictating data integrity between these settings. While Alghamdi attributed incompleteness in primary care to the transition from paper to electronic records, our study—situated in a fully digital tertiary infectious disease center—identified clinical acuity as the differentiator. Unlike the primary care setting, where chronic disease management drives documentation, our data suggests that the urgent nature of infectious disease admissions (short LOS) necessitates rapid, comprehensive data entry for isolation protocols, a factor absent in the primary care literature [16]. These findings suggest that organizational factors, such as staffing stability and robust, continuous training programs, are crucial for improving data quality.

Our analysis revealed that data completeness was significantly higher in inpatient encounters (55.8%) compared to outpatient encounters (44.2%). This is likely because the extended duration of inpatient stays provides clinicians with more opportunities for thorough patient evaluation and documentation. This observation aligns with findings from Weiskopf et al., who also reported higher completeness rates for inpatient data [20]. However, it contrasts with the findings of a study from Basrah, which found poorer documentation in inpatient settings, suggesting that local workflow priorities, such as a focus on procedural interventions over documentation, can override the influence of encounter type [31].

The implementation of CDSSs appears to positively influence documentation. In our study, 70% of complete records had at least one alert notification. Mechanistically, this association is likely driven by the interdependency of CDSSs features; safety checks such as standardized drug dosages and drug–drug interaction warnings cannot be triggered unless foundational data (e.g., patient weight and allergy status) are entered first. Therefore, the alert system effectively acts as a forcing function, compelling clinicians to input ‘breadth’ variables to enable these safety tools. While our analysis focused on the presence of alerts, this finding supports the premise that CDSSs integration indirectly mandates higher data fidelity. However, it is important to distinguish that this completeness correlates with the presence of alerts. Future analysis should aim to stratify the impact of specific alert types (e.g., interruptive ‘hard stops’ for missing data vs. passive advisory alerts) to determine which specific mechanisms most effectively prevent data omission [32,33]. Furthermore, the involvement of clinical pharmacists in medication reconciliation was associated with higher completeness in this specific domain (34.6%), underscoring the value of interdisciplinary collaboration in the data entry process.

Interestingly, a longer length of stay was associated with a higher likelihood of incomplete records, particularly for stays exceeding seven days (24.5% incomplete vs. 75.5% complete for shorter stays). This inverse relationship may be attributed to the clinical workflow specific to a tertiary referral center specializing in infectious diseases. Patients with shorter stays often present with acute conditions requiring immediate, comprehensive data entry (height, weight, and allergies) to calculate precise anti-infective dosing and determine isolation protocols upon admission. Consequently, the ‘admission phase,’ which demands high data fidelity, represents a significant portion of a short stay. Conversely, for patients with prolonged hospitalizations, the volume of daily progress notes increases, yet the repetition of static data (such as anthropometrics) is not required, potentially leading to gaps in daily documentation frequency as clinicians focus on monitoring rather than initial data capture.

The COVID-19 pandemic introduced unique stressors to CPOE usage intensity that likely influenced our 2019–2021 data trends. The rapid shift to remote workflows and the establishment of tele-ICU services necessitated ‘remote order entry,’ a process distinct from bedside entry. Almazrou et al. (2021) analyzed medication errors during this period in Saudi Arabia and found that ‘missing clinical information’ (e.g., weight, allergies) accounted for nearly 15% of reported errors, suggesting that the physical separation of the prescriber from the patient in remote workflows may exacerbate data omission [26]. Furthermore, a systematic review by Alsahmah et al. (2024) on digital transformation in Saudi Arabia noted that while remote tools improved access [34], they often bypassed the forced-function alerts typical of inpatient desktops, leading to variations in data integrity [34]. This context suggests that the ‘improvement’ we observed in 2019 may have been blunted in subsequent years by pandemic-related workflow disruptions, a variable that future longitudinal studies must isolate.

### 4.2. Implications for Practice and Research

The findings underscore the critical need for multifaceted strategies to improve CPOE data quality. Our analysis revealed that vital signs, allergies, and weight were missing in approximately 50% of records. To rectify this, hospital administrators should configure the CPOE system to utilize ‘Hard Stop’ constraints for these specific variables. For instance, the system should prevent the finalization of an admission profile until height and weight are recorded, as these are critical for weight-based dosing. Furthermore, given that 70% of complete records in our study were associated with alert notifications, we recommend implementing ‘Just-in-Time’ interruptive alerts. Rather than passive notifications, these alerts would block a physician from signing a medication order if the ‘Allergy’ field is blank, thereby forcing immediate compliance without causing alert fatigue during non-prescribing tasks.

The stark contrast in completeness between short stays (<7 days, 75.5% complete) and long stays (>30 days, 2% complete) indicates a failure in data maintenance after the initial admission workup. For patients with prolonged hospitalizations, the initial data often becomes obsolete. We propose the implementation of a ‘Weekly Data Refresh’ protocol within the EHR. This would involve an automated task generating a ‘soft alert’ for nursing staff every 7 days, prompting a re-evaluation of weight and vital signs. This ensures that the CDSSs continue to operate on current data, preventing the degradation of data quality that was observed in our long-stay cohort.

Finally, since medication reconciliation by pharmacists was associated with a 2.5-fold increase in data completeness, this workflow should be formalized. We suggest granting clinical pharmacists ‘editor’ privileges for demographic and anthropometric fields, allowing them to fill in gaps in weight or allergy history during their reconciliation process, thereby sharing the documentation burden across the multidisciplinary team.

### 4.3. Future Directions

Based on the study findings, several future directions are recommended to mitigate the risks associated with incomplete documentation and improve healthcare outcomes. To enhance data completeness, future efforts should focus on technical and organizational interventions. Furthermore, implementation of “hard stops” or mandatory fields in the CPOE system for critical anthropometric data (height and weight) and vital signs can ensure that these are not bypassed. In addition, to mitigate “alert fatigue,” future system updates must refine CDSSs triggers to be highly specific and sensitive, ensuring that clinicians are only prompted when necessary. Moreover, regular training programs should be instituted to address the high turnover of residents and locum staff to ensure that new providers are proficient in documentation protocols immediately upon hiring.

While this study did not directly measure monetary costs, the link between data completeness and financial impact was established through patient safety. Incomplete data compromises the CPOE system’s ability to prevent Adverse Drug Events (ADEs). The literature indicates that preventable ADEs are a significant financial burden on healthcare systems [35]. By resolving documentation gaps—specifically in weight and allergies—hospitals can maximize the CPOE system’s ability to prevent these errors, thereby reducing the substantial costs associated with prolonged hospitalizations and treatments for medication errors. Future research should aim to quantify the direct cost-savings realized by achieving 100% data completeness in this setting.

### 4.4. Strengths and Limitations

A key strength of this study is its use of multiple definitions of completeness to analyze data from three distinct years, providing a nuanced and longitudinal perspective on CPOE data quality. Moreover, this study offers two distinct contributions to the literature on health informatics in Saudi Arabia. First, it identifies a novel correlation between length of stay (LOS) and data integrity specifically within an infectious disease cohort. Contrasting with the assumption that longer hospitalizations allow for more thorough documentation, we identified a ‘completeness decay’ in long-term patients, highlighting a critical vulnerability in the maintenance phase of electronic records. Second, this study is among the first in the region to quantify the operational value of pharmacist-led medication reconciliation in a CPOE environment. By verifying that pharmacist involvement increases the odds of data completeness by 2.5 times, we provide empirical evidence validating the expansion of clinical pharmacy roles in Saudi hospitals, not just for medication safety, but as guardians of essential health data quality.

However, the study has several limitations. First, the findings are derived from a single tertiary care referral center specializing in infectious diseases (including MERS-CoV and COVID-19). Consequently, the results may not be generalizable to non-infectious disease hospitals, community health service centers, or outpatient clinics with lower acuity levels and different documentation workflows. The high-stress environment and strict isolation protocols inherent to this facility may introduce unique barriers to documentation that are not present in general hospital settings. Therefore, our conclusions regarding CPOE completeness are most applicable to similar high-complexity, tertiary acute care environments rather than primary care settings.

Additionally, the exclusion of data from 2016 and 2018—while necessary to remove artifacts caused by system upgrades and staff adaptation periods—means that the study presents a snapshot of progress rather than a continuous timeline. Furthermore, while we identified a strong association between alert notifications and data completeness, this study analyzed alerts as a binary variable (presence/absence). We were unable to stratify the analysis by specific alert types (e.g., drug allergy warnings vs. administrative missing-data prompts) or trigger frequency, which limits our ability to identify which specific alert mechanisms are most effective at driving documentation compliance. Future multi-center studies including general hospitals are needed to validate these trends universally.

Finally, while we controlled for patient-level factors, our analysis did not account for clustering by individual prescriber, as provider IDs were anonymized at the source; therefore, the analysis could not adjust for clustering by prescriber using mixed-effects models. We acknowledge that this may lead to an underestimation of standard errors if documentation habits are highly correlated within specific physicians.

## 5. Conclusions

The completeness of CPOE data is highly dependent on the specific definition used and the clinical information deemed essential. While this study revealed a positive trend of improvement in data completeness over time, a significant proportion of crucial clinical data remains missing. It is important to note that these findings are derived from a specialized tertiary center focusing on infectious diseases (including MERS-CoV and COVID-19). Consequently, while the positive trends indicate that system maturity improves documentation even in high-pressure environments, the results may vary in non-specialized or community health service centers where workflows differ. This information gap poses a potential risk to patient safety and limits the utility of electronic health records for research and quality improvement. Achieving comprehensive documentation requires a sustained, organization-wide commitment to optimizing system design, providing continuous staff training, and fostering a culture that values high-quality data as an integral component of patient care. Future multi-center studies are recommended to validate these trends across a broader spectrum of healthcare institutions.

## Figures and Tables

**Table 1:** Patient baseline characteristics.

Characteristic	Year			*p*-Value
	2015 (N = 200)	2017 (N = 200)	2019 (N = 200)	
**Age, (mean ± SD)**	38.62 ± 18.59	46.46 ± 16.93	50.38 ± 16.13	<0.0001 *
**Number of prescription medications, (mean ± SD)**	5.525 ± 2.62	4.58 ± 2.57	5.05 ± 2.18	<0.0001 *
**Gender**				
Female, n (%)	81 (40.50)	82 (41.00)	76 (38.00)	0.806
Male, n (%)	119 (59.50)	118 (59.00)	124 (62.00)	
**Marital status**				
Married n (%)	120 (60.00)	148 (74.00)	153 (76.50)	0.0005 *
Single, n (%)	80 (40.00)	52 (26.00)	47 (23.50)	
**Patient encounter**				
Inpatient, n (%)	95 (47.50)	125 (62.50)	104 (52.00)	0.009 *
Outpatient, n (%)	105 (52.50)	75 (37.50)	96 (48.00)	
**Length of stay, (mean ± SD)**	6.84 ± 6.89	9.23 ± 7.66	7.86 ± 7.20	0.0049 *
**Alert, n (%)**	102 (51.00)	118 (59.00)	148 (74.00)	<0.0001 *

* *p* < 0.05.

**Table 2:** Frequency and proportion of missing data per variable across study years.

Variable	Year			*p*-Value	Effect Size (Cramer’s V)
	2015 (N = 200) N, Missing (%)	2017 (N = 200) N, Missing (%)	2019 (N = 200) N, Missing (%)		
**Demographics**					
**Age**	0 (0%)	0 (0%)	0 (0%)	-	-
**Gender**	0 (0%)	0 (0%)	0 (0%)	-	-
**Marital status**	0 (0%)	0 (0%)	0 (0%)	-	-
**Anthropometrics**					
**Weight, n (%)**	148 (74.0%)	119 (59.5%)	14 (7.0%)	<0.0001 *	0.5708
**Height, n (%)**	153 (76.5%)	143 (71.5%)	23 (11.5%)	<0.0001	0.6480
**Clinical Data**					
**Vital signs, n (%)**	148 (74.0%)	140 (70.0%)	55 (27.5%)	<0.0001 *	0.7026
**Diagnosis, n (%)**	64 (32.0%)	36 (18.0%)	21 (10.5%)	<0.0001 *	0.3057
**Allergies, n (%)**	170 (85.0%)	90 (45.0%)	54 (27.0%)	<0.0001 *	0.6372
**Overall** **missing** **data at patient level, n (%)**	189 (94.50)	148 (74.00)	101 (50.50)	<0.0001 *	

* *p* < 0.05.

**Table 3:** Odds ratio of bivariate regression analysis for having complete data in the CPOE system.

Variable	Odds Ratio (OR)	95% Confidence Interval (CI)		*p*-Value
		Lower	Upper	
Year (2017 vs. 2015)	6.04	3.04	11.98	<0.0001 *
Year (2019 vs. 2015)	16.84	8.63	32.85	<0.0001 *
Length of stay	1.04	1.01	1.06	0.0023 *
Gender	1.09	0.75	1.57	0.6427
Medication reconciliation	2.51	1.73	3.64	<0.0001 *
Admission	1.29	0.899	1.86	0.165
Inclusivity of data due to alert	1.937	1.308	2.868	0.001 *
Number of prescription medications	1.068	0.995	1.146	0.066

* *p* < 0.05.

**Table 4:** Multiple logistic regression analysis for the impact of time (year) on the completeness of data for CPOE.

Variable	Odds Ratio (OR)	95% Confidence Interval (CI)		*p*-Value
		Lower	Upper	
**Year (2017 vs. 2015)**	6.18	2.93	13.03	<0.0001 *
**Year (2019 vs. 2015)**	17.47	8.25	37.01	<0.0001 *
**Age**	0.99	0.982	1.006	0.292
**Length of stay**	1.04	1.001	1.070	0.045 *
**Gender**	1.28	0.84	1.97	0.257
**Medication reconciliation**	1.15	0.75	1.77	0.519
**Admission Type (Inpatient)**	0.87	0.54	1.40	0.563
**Number of prescription medications**	1.08	0.99	1.19	0.091

* *p* < 0.05.

## Data Availability

The data presented in this study are available on request from the corresponding author due to legal restrictions under the Personal Data Protection Law in Saudi Arabia. However, aggregate contingency tables containing the frequencies used for statistical analyses are provided in Supplementary Tables S1 and S2. Further inquiries regarding data availability may be directed to the corresponding author.

## Supplementary Materials

The following supporting information can be downloaded at: https://www.mdpi.com/article/10.3390/healthcare14020179/s1, Table S1: Aggregate Contingency Table for Patient Baseline Characteristics by Year; Table S2: Aggregate Contingency Table for Data Completeness (“Breadth”) by Year.
