# Supplementary material for: Evolution of Computerized Provider Order Entry Documentation at a Leading Tertiary Care Referral Center in Riyadh
Source: Healthcare (Basel). 2026 Jan 10;14(2):179. doi: 10.3390/healthcare14020179 (PMC12841437; doi:10.3390/healthcare14020179)
Supplement: Supplementary file 1 [file healthcare-14-00179-s001.zip › healthcare-3978244-supplementary.pdf]

Table S1. Aggregate Contingency Table for Patient Baseline Characteristics by Year.

Variable	Category	2015 (N=200) N (%)	2017 (N=200) N (%)	2019 (N=200) N (%)	Total (N=600) N (%)	*P*-value
**Gender**	Male	81(40.50)	82(41.0)	76(38.0)	239(39.83)	0.806
	Female	119(59.50)	118(59.0)	124(62.0)	361(60.16)	
**Marital status**	Married	120(60.0)	148(74.0)	153(76.5)	421(70.16)	0.0005*
	Single	80(40.0)	52(26.0)	47(23.5)	179(29.83)	
**Encounter type**	Inpatient	95(47.5)	125(62.5)	104(52)	324(54)	0.009*
	Outpatient	105(52.5)	75(37.5)	96(48)	276(46)	
**Length of Stay**	<7 days	168(84.0)	132(66.0)	153(76.5)	453(75.5)	0.0003*
	7-30 days	28(14.0)	65(32.5)	43(21.5)	136(22.66)	
	>30 days	4(2.0)	3(1.5)	4(2.0)	11(1.83)	
**Alert Presence**	Yes	102(51.0)	118(59.0)	148(74.0)	368(61.33)	<.0001*
	No	98(49)	82(41.0)	52(26.0)	232(38.66)	

*$P<0.05$.

Table S2. Aggregate Contingency Table for Data Completeness ("Breadth") by Year.

Variable (Complete Data)	2015 (N=200) N (%)	2017 (N=200) N (%)	2019 (N=200) N (%)	Total (N=600) N (%)	*P*-value
**Weight**	52(26)	81(40.5)	186(93.0)	319(53.16)	<0.0001
**Height**	47(23.5)	57(28.5)	177(88.5)	281(46.83)	<0.0001
**Vital Signs**	52(26.0)	60(30.0)	145(72.5)	257(42.83)	<0.0001
**Diagnosis**	136(68.0)	164(82.0)	179(89.5)	479(79.83)	<0.0001
**Allergies**	30(15.0)	110(55.0)	146(73.0)	286(47.66)	<0.0001
**Overall Complete (All Variables)**	11(5.5)	52(26.0)	99(49.5)	162(27.0)	<0.0001
